# 20 Hz tACS engages striatal THINs to enhance β-band synchrony and motor drive

**DOI:** 10.3389/fnins.2026.1887979

**Published:** 2026-09-04

**Authors:** Honggang Zang, Wanxia Xie, Lei Tian, Wanrong Kang, Yulan Li

**Affiliations:** 1 The First School of Clinical Medicine, Lanzhou University, Lanzhou, China; 2 Department of Ultrasound Medicine, Gansu Provincial Hospital, Lanzhou, China; 3 Gansu University of Chinese Medicine, Lanzhou, China

**Keywords:** aging, anesthesia, motor function, tACS, THINs

## Abstract

**Background:**

Transcranial alternating current stimulation (tACS) is a noninvasive neuromodulation technique that can enhance motor function. The striatum is a central hub for motor control and β-band oscillations, and its tyrosine hydroxylase-positive interneurons (THINs) are modulated by dopamine. However, whether THINs are associated with motor changes following tACS through β-band synchrony has not been investigated.

**Methods:**

Eighteen-month-old male mice received 20 Hz tACS or sham stimulation over M1 for 7 days under sevoflurane anesthesia. Motor performance, EEG, striatal dopamine release, and THIN activation were assessed at 3 min after awakening on day 7. THINs were optogenetically manipulated during the reaching task under the same post-anesthesia testing window.

**Results:**

Transcranial alternating current stimulation was associated with higher post-anesthesia reaching success, increased β-band power, elevated dopamine release, and enhanced THIN activation compared to sham. Optogenetic THIN activation recapitulated motor improvement and β-band enhancement. Optogenetic THIN inhibition reduced successful attempts per minute and β-band power, but no statistically significant reduction in success rate was detected.

**Conclusion:**

These findings suggest that 20 Hz tACS over M1 may improve post-anesthesia motor performance in aged mice, with converging evidence implicating striatal dopamine release, THIN engagement, and β-band synchrony as functionally coupled elements in the context of post-anesthesia motor recovery in aged mice.

## 1 Introduction

Transcranial alternating current stimulation is a rapidly evolving noninvasive neuromodulation technique. Its core principle relies on applying weak alternating currents at specific frequencies to entrain endogenous brain oscillations through resonance-like effects, thereby modulating brain function. For instance, a 3-weeks tACS intervention was shown to significantly improve cognitive function in patients with mild Alzheimer’s disease compared with sham stimulation, an effect attributed to enhanced functional connectivity (Wang et al., 2025). In the motor domain, similar frequency-specific effects have been proposed (McNally et al., 2024), yet the underlying circuits remain incompletely understood.

The primary motor cortex (M1) and the striatum together form the core of the cortico-basal ganglia loop that orchestrates the initiation and execution of movement (Yang et al., 2025; Nicholas and Yttri, 2024). However, the role of the striatum in motor changes observed with tACS has not been clearly defined. Elucidating this role requires consideration of the striatum’s unique cellular and plasticity features. The striatum is composed of projection neurons and interneurons, with projection neurons accounting for 90%–96% and interneurons for 4%–10% of the neuronal population (Prager and Plotkin, 2019; Hjorth et al., 2020). It receives one of the densest dopaminergic innervations in the brain, originating exclusively in the striatum, and serves as a central hub where dopamine-dependent synaptic plasticity has been extensively characterized–a property critical for motor learning, corticostriatal integration, and motor control (Stiers and Goulas, 2022; Bech et al., 2023; Salvatore, 2024). Importantly, the striatum has also been identified as a key downstream target of oscillatory–plasticity coupling (Banaie Boroujeni and Womelsdorf, 2023; Lemke et al., 2021). Among striatal interneurons, tyrosine hydroxylase-positive interneurons (THINs) are sparse in number yet functionally important (Assous et al., 2017); despite expressing dopaminergic markers, these interneurons neither synthesize nor release dopamine but instead exert powerful feedforward inhibition onto medium spiny neurons (MSNs) via GABAA receptors (Ibáñez-Sandoval et al., 2015; Xenias et al., 2015), and their activity is itself modulated by dopamine (Ibáñez-Sandoval et al., 2015).

The striatum is intimately associated with β-band oscillations, which are widely implicated in motor and cognitive processing (Engel and Fries, 2010; Siegel et al., 2009; Varela et al., 2001). During tasks that demand cognitive control, β-band activity between the frontal cortex and striatum is modulated, indicating that β rhythms participate in goal-directed behavior (Koloski et al., 2024). Whether and how 20 Hz tACS is temporally correlated with striatal THIN activity, and whether this activity correlates with motor performance, remain unknown. However, whether these correlational observations reflect a causal sequence remains to be determined. To address these questions, we delivered 20 Hz tACS (0.15 mA) over the M1 of aged mice under controlled sevoflurane anesthesia.

## 2 Materials and methods

### 2.1 Ethical statement, animal model, and study design

All animal procedures were conducted in accordance with the National Institutes of Health Guide for the Care and Use of Laboratory Animals and approved by the Institutional Animal Care and Use Committee of Lanzhou University (Protocol No. LDYYLL2025-44). This study is reported in compliance with the ARRIVE 2.0 guidelines.

Eighteen-month-old male C57BL/6J mice (26–31 g) were used in this study. All mice were sourced from the Lanzhou Veterinary Research Institute of the Chinese Academy of Agricultural Sciences. The use of a single-sex cohort precluded potential confounds from estrous cycle hormone fluctuations on motor and neural plasticity outcomes. Mice were acclimatized for 4 weeks under specific pathogen-free conditions (23 °C ± 1 °C, 12-h light/dark cycle, lights on at 07:00). To minimize circadian influences, all procedures were performed during the active phase of the animals (after 19:30).

### 2.2 Single-pellet reaching task: training and assessment

Motor function was assessed using the single-pellet reaching task. The mice were food-restricted to maintain >90% of their baseline body weight to ensure their motivation, outside the daily food restriction periods, mice had *ad libitum* access to standard chow and water. Training spanned 14–16 days and comprised habituation, shaping (5–7 days), and formal training (7 days). During shaping, the mice learned to reach corn pellets with the right forelimb through a narrow slit. Only mice that achieved a success rate > 40% with the right forelimb (successful retrieval and consumption without dropping) by the end of shaping were included in subsequent experiments, ensuring a stable baseline of skilled motor performance. The single-pellet reaching task was performed at two time points: on the second day prior to the first stimulation (baseline) and at 3 min after awakening on day 7, awakening was defined as the recovery of the righting reflex. All post-stimulation behavioral tests (including the single-pellet reaching task and EEG recordings) were performed 3 min after righting reflex recovery.

#### 2.2.1 Surgical procedures for electrode implantation

The mice were anesthetized with 2.3% sevoflurane and placed in a stereotaxic frame. After a scalp incision and skull leveling, burr holes were drilled at the following coordinates relative to bregma: for transcranial alternating current stimulation (tACS) of the primary motor cortex (M1: AP +1.5 mm, ML +2.2 mm); for the positive EEG recording electrode (AP +1.5 mm, ML +0.5 mm); for the negative EEG recording electrode (AP −0.5 mm, ML −0.5 mm); and for a reference electrode (AP +3 mm, ML +1.5 mm). Custom-made screw electrodes were implanted, and the assembly was secured with dental acrylic. After surgery, mice were singly housed and allowed to recover for 7 days with *ad libitum* food and water before any further procedures.

#### 2.2.2 Transcranial alternating current stimulation protocol

Mice were randomly assigned to the tACS or sham group (*n* = 8). The tACS group was subjected to 20 Hz sinusoidal current at 0.15 mA over the left M1 for 20 min daily, administered 2.3% sevoflurane anesthesia over seven consecutive days. The sham group underwent an identical procedure, including anesthesia, but did not receive current stimulation. On day 7, after the final tACS (or sham) session, the mice were awakened and tested on the single-pellet reaching task. All animals tolerated the repeated anesthesia well, with no signs of distress or weight loss, and the cohort included the sham and tACS groups, each with corresponding baseline conditions (B-Sham and B-tACS).

#### 2.2.3 EEG

EEG signals were recorded from awake mice using screw electrodes implanted epidurally. The active electrode was placed over the left M1 (AP +1.5 mm, ML +0.5 mm relative to bregma), the reference electrode over the frontal bone (AP +3 mm, ML +1.5 mm), and the ground electrode over the cerebellum. Signals were amplified (×1000), band-pass filtered at 0.4–80 Hz, and digitized at a sampling rate of 2000 Hz (PowerLab 16/35, ADInstruments). A 50 Hz notch filter was applied offline. Recordings were performed at baseline (2 days before the first stimulation) and at 3 min after awakening on day 7. Relative power was estimated using Welch’s method (Hamming window, 1-s windows, 50% overlap) in MATLAB R2024. Relative power was calculated for each frequency band: delta (0.5–4 Hz), theta (4–8 Hz), alpha (8–12 Hz), beta (12–30 Hz), and gamma (30–80 Hz).

#### 2.2.4 FSCV for striatum dopamine release

Calibration is performed using fast-scan cyclic voltammetry (FSCV) under the following parameters: potential range from −0.4 V to +1.3 V, scan rate of 400 V/s, scan frequency of 10 Hz, and holding potential of −0.4 V. Dopamine standard solution was added sequentially to artificial cerebrospinal fluid (aCSF) at pH 7.2 with 20 mg/mL bovine serum albumin (BSA) to obtain five concentrations (0, 0.05, 0.5, 1, and 5 μmol/L). For each concentration, the average oxidation peak current was extracted from the 190th to the 200th scan as the ordinate (Y), and plotted against the corresponding dopamine concentration as the abscissa (X), thereby constructing a standard curve from which a linear regression equation is derived. Electrodes demonstrating a linear response with a correlation coefficient (R^2^ > 0.99) and a sensitivity exceeding 40 nA/μM were selected for subsequent *in vivo* recordings. Figure supplement 1 is a typical response of the electrode to physiological concentrations of dopamine.

Under sevoflurane anesthesia used for tACS delivery, a carbon-fiber microelectrode was implanted into the striatum (AP +1.5 mm, ML +1.2 mm, DV −2.6 mm) to monitor dopamine release. The tACS electrode (M1) delivered the 20 Hz sinusoidal current (0.15 mA), and dopamine concentration was continuously measured using a triangular waveform (−0.4 V to +1.3 V, 400 V/s, applied every 100 ms). The background subtraction were performed with MT FSCV software (MT Technology). Specifically, the background cyclic voltammogram was defined as the average of ten FSCV cycles recorded prior to electrical stimulation. Subsequent raw cyclic voltammogram data obtained after the stimulus were then processed by subtracting this averaged background template. Background-subtracted cyclic voltammograms were obtained, and dopamine was identified by its oxidation peak at ∼+0.6 V. each animal underwent identical procedures but without current delivery. After a 1-week recovery from electrode implantation, striatal dopamine levels were measured in response to sham stimulation and 20 Hz tACS delivered via the stimulating electrode. To allow each animal to serve as its own control, sham stimulation (without current delivery) was first applied, followed by the delivery of 20 Hz tACS (0.15 mA) via the stimulating electrode on the same individual. Dopamine concentrations were continuously monitored throughout both conditions.

#### 2.2.5 Immunofluorescence for THINs activation

On day 7, after the final tACS (or sham) session, the mice were deeply anesthetized and perfused with saline, followed by 4% paraformaldehyde. Paraffin-embedded coronal sections were deparaffinized and rehydrated through sequential incubations in environmentally friendly dewaxing solution (three changes, 10 min each) and absolute ethanol (three changes, 5 min each), followed by rinsing with distilled water and heat treatment for 30 min. After retrieval, sections were cooled to room temperature and washed three times (5 min each) in PBS (pH 7.4) on a shaker. For double labeling, c-fos+ and Tyrosine Hydroxylase antibodies raised in two different species were mixed (GB12069/GB150057, Servicebio, both at 1:600 dilution) and applied to sections. Sections were incubated overnight at 4 °C in a humidified chamber. After three times in PBS (5 min each), sections were incubated with species-appropriate fluorophore-conjugated secondary antibodies for 50 min at room temperature in the dark. Nuclei were counterstained with DAPI for 10 min at room temperature in the dark. To suppress tissue autofluorescence, sections were incubated with autofluorescence quenching reagent B for 5 min, followed by a 10 min tap water rinse, coverslipped with anti-fade mounting medium, images were acquired using a fluorescence microscope, and at least three sections per animal were quantified.

#### 2.2.6 Viral injection and optic fiber implantation

Mice received stereotaxic injections of either a control virus (rAAV-TH-EGFP) or an experimental virus (rAAV-TH-hChR2(H134R)-EGFP; 300 nL at 20 nL/min) into the striatum (AP +1.5 mm, ML +1.5 mm, DV −2.6 mm). The TH promoter drives transgene expression in cells that actively transcribe TH. In the striatum, intrastriatal injection of TH-promoter-dependent viruses selectively transduces striatum-intrinsic THINs with no detectable transduction of midbrain dopaminergic neurons or their terminals. All viral reagents were purchased from the brainVTA.

Optogenetic stimulation and histology. For optogenetic activation, mice received intrastriatal injections of either a control virus (rAAV-TH-EGFP) or a channelrhodopsin-2 virus (rAAV-TH-hChR2(H134R)-EGFP) and were subdivided into no-light or blue-light conditions, yielding groups C, C-Blue, L-Sham, and L-Blue. For optogenetic inhibition, a separate cohort was injected with the same control virus or a halorhodopsin virus (rAAV-TH-eNpHR3.0-EGFP) and similarly assigned to no-light or yellow-light conditions, forming groups C, C-Yellow, L, and L-Yellow. Three weeks after viral injection, during the single-pellet reaching task, mice in the activation groups received 470 nm blue light pulses (20 Hz, 5 ms pulse width, 10 mW) in a 2 s on/5 s off cycle, while mice in the inhibition groups received continuous 560 nm yellow light (8 mW) for the entire 20 min stimulation period. Optogenetic stimulation was delivered at 3 min after recovery of the righting reflex following sevoflurane anesthesia (2.3%), i.e., under the same post-anesthesia testing window as used for the tACS behavioral assessments (see Section “2.2.2 Transcranial alternating current stimulation protocol”). Mice were awake and freely moving during the single-pellet reaching task, with optical stimulation delivered concurrently with behavioral and EEG. Immediately after stimulation, animals were transcardially perfused with 0.1 M phosphate-buffered saline (PBS) followed by 4% paraformaldehyde (PFA). Brains were removed, post-fixed overnight in 4% PFA at 4 °C, and sectioned coronally at 50 μm thickness throughout the striatum using a vibrating microtome (VT2000, Leica). Free-floating sections were blocked in PBS containing 0.1% bovine serum albumin (BSA), 2% normal goat serum (NGS), and 0.3% Triton X-100 for 2 h at room temperature. After washing, sections were mounted onto gelatin-coated slides, coverslipped with DAPI-containing mounting medium, and imaged using a confocal laser scanning microscope (LSM 710, Zeiss) with a 20× objective. For quantitative colocalization analysis of viral specificity, three animals per viral construct (ChR2 and eNpHR3.0) were used. Three coronal sections spanning the rostrocaudal extent of the striatum were analyzed per animal. The number of EGFP-positive neurons and TH-EGFP double-positive neurons was manually counted in a blinded manner across all sections.

### 2.3 Randomization, blinding, and exclusion criteria

Mice were randomly assigned to experimental groups using a computer-generated random sequence. The experimenter performing behavioral scoring, EEG preprocessing, and histological quantification was blinded to group allocation. The investigator delivering tACS or optogenetic stimulation was different from the one conducting the outcome assessments. Exclusion criteria were predefined before the start of the experiment: animals that failed to reach > 40% success rate at the end of shaping or that showed signs of illness or weight loss > 15% during the experimental period were excluded from the final analysis. All excluded animals were replaced with new mice to maintain the planned sample size. The final sample sizes for each group are reported in the figure legends and in the section “3 Results.”

### 2.4 Statistical analysis

Data are presented as the mean ± SEM. Normality and homogeneity of variance were assessed using Shapiro-Wilk and Levene’s tests, respectively. For comparisons involving two independent groups, unpaired two-tailed Student’s *t*-tests (parametric) or Mann-Whitney U tests (non-parametric) were used. For repeated-measures data (multi-band EEG power), repeated-measures analysis of variance (RM-ANOVA) with Greenhouse–Geisser correction for sphericity violations was applied. *Post hoc* pairwise comparisons were performed using Bonferroni-corrected *t*-tests to control the family-wise error rate across multiple frequency bands and experimental conditions. A value of *P* < 0.05 was considered statistically significant. Mice were randomly assigned to groups using a computer-generated sequence, and experimenters performing behavioral scoring, EEG preprocessing, and histological quantification were blinded to group allocation. All analyses were conducted using SPSS 29.0, and graphs were generated using GraphPad Prism 10 and Origin 2021.

## 3 Results

### 3.1 20 Hz tACS over M1 enhances skilled reaching performance in aged mice under sevoflurane anesthesia

**FIGURE 1:**
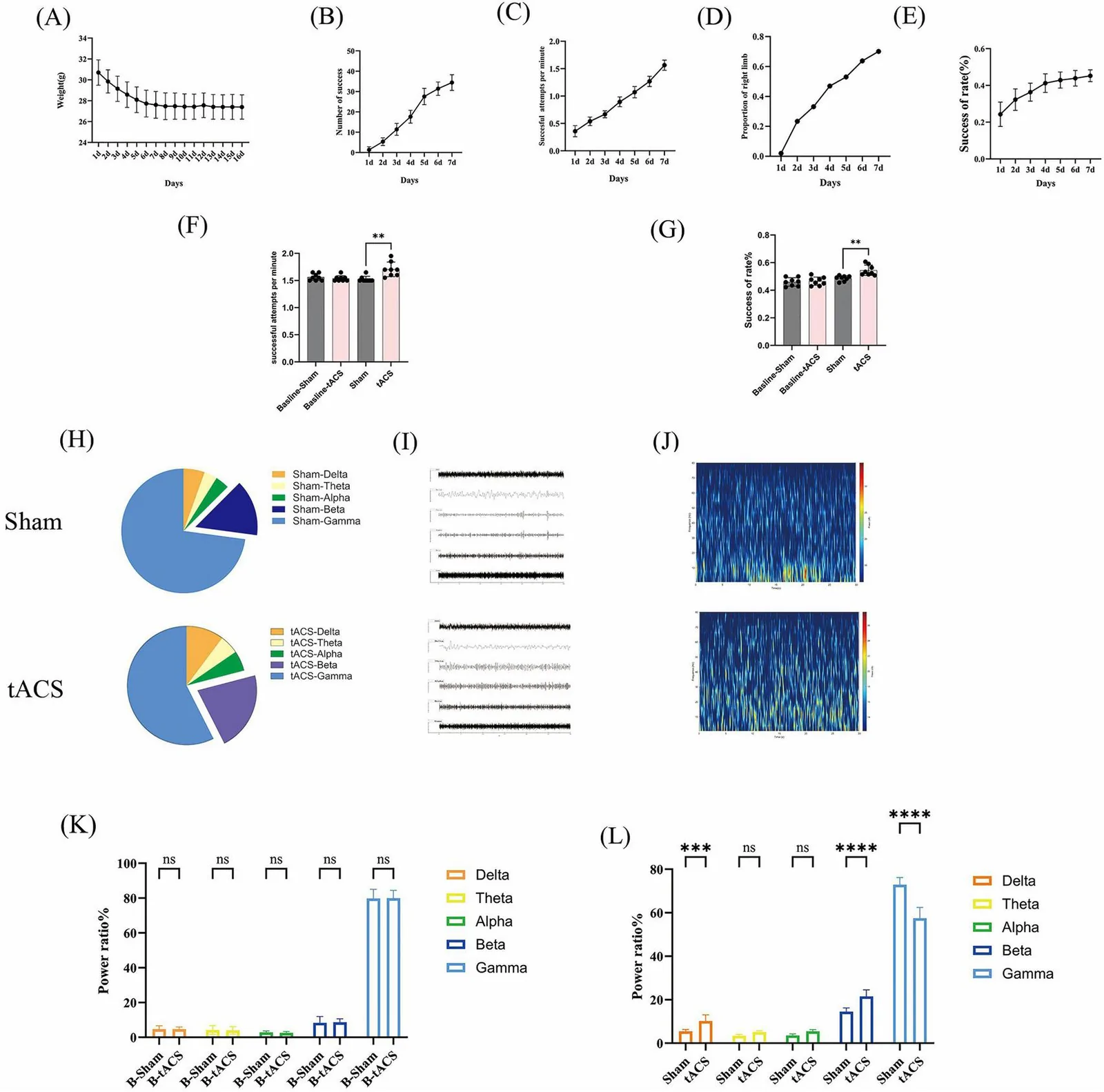
Behavioral and EEG outcomes following sevoflurane alone or combined with tACS. **(A)** Changes in animal body weight during training; **(B)** The number of attempts the mice made to complete the task during the plasticity phase; **(C)** Proportion of right upper limb use during the plasticity stage; **(D)** The speed at which tasks are successfully executed during the training phase; **(E)** Success rate of task completion during the training phase; **(F)** Baseline the successful attempts per minute (B-Sham, B-tACS) were recorded prior to any intervention; Post-intervention success rates were assessed on day 7 following sevoflurane inhalation alone (Sham) or combined sevoflurane and tACS treatment (tACS), (*n* = 8 per group); **(G)** Baseline the success rate (B-Sham, B-tACS) were recorded prior to any intervention. Post-intervention success rates were assessed on day 7 following sevoflurane inhalation alone (Sham) or combined sevoflurane and tACS treatment (tACS), (*n* = 8 per group); **(H)** Pie charts showing the overall distribution of relative power (percentage of total power) across standard EEG frequency bands (δ, θ, α, β, γ) in Sham and tACS groups at the end of the experiment. **(I)** Representative preprocessed EEG waveforms; **(J)** Corresponding time–frequency plots for each condition; **(K)** Bar graphs illustrating changes in relative EEG power at baseline (B-Sham, B-tACS); **(L)** Bar graphs illustrating changes in relative EEG power at after 7 days of sevoflurane exposure without or with tACS (Sham, tACS). Statistical analysis compared with the Sham group, **p < 0.01, ***p < 0.001, ****p < 0.0001.

During the 16-days training period, all mice met the prespecified criteria: body weight remained >90% of baseline, they achieved >20 successful trials in the 7-days shaping phase with >70% right-forelimb preference, and maintained a task performance of >1.5, successful trials/min with >40% success rate. The detailed data are provided in Figures 1A–E. At baseline, there was no significant difference in the success rate between the group subsequently assigned to the sham and the group assigned to the tACS (B-Sham vs. B-tACS, 95% CI [−0.0267, 0.0380], *p* = 7135, *t* = 0.3746, *n* = 8 per group; Figures 1F, G). After 7 days of continuous 20-min tACS over the left M1 region, the successful attempts per minute in the tACS group increased significantly (1.71 ± 0.13 vs. 1.50 ± 0.05 in the sham group, *p* = 0.0011, *U* = 3.5; Figure 1F). and the success rate increased significantly (54.46% ± 5.81% vs. 48.65% ± 1.50% in the sham group, *p* = 0.0016, *t* = 3.886, df = 14; Figure 1G). Time-frequency analysis confirmed a distinct power elevation centered at 20 Hz (Figures 1I, J). At baseline, there was no significant difference in the δ(95% CI [−2.806, 2.865], *p* = 09835, *n* = 8 per group), θ(95% CI [−2.691, 2.979], *p* = 0.9196, *n* = 8 per group), α(95% CI [−2.549, 3.122], *p* = 0.8409, *n* = 8 per group), β(95% CI [−3.121, 2.550], *p* = 0.8412, *n* = 8 per group), γ(95% CI [−3.010, 2.661], *p* = 0.9029, *n* = 8 per group) band intensity between the group subsequently assigned to sham and the group assigned to tACS (B-Sham vs. B-tACS, *p* > 0.05; Figure 1K). EEG spectral analysis revealed that tACS significantly increased the relative power in the δ(10.25% ± 2.80% vs. 5.49% ± 0.83%, *p* = 0.0002, *t* = 3.994, 95% CI [−7.139, −2.384]), β(21.58% ± 3.00% vs. 14.60% ± 1.64%, *p* = 0.0001, *t* = 5.856, 95% CI [−9.358, −4.603]), and reduction the relative power in the γ(57.48% ± 4.94% vs. 72.87% ± 3.27%, *p* = 0.0001, *t* = 12.920, 95% CI [13.02, 17.77]) band compared to sham stimulation (*p* < 0.05; Figure 1L).

### 3.2 tACS evokes phasic dopamine release in the striatum

**FIGURE 2:**
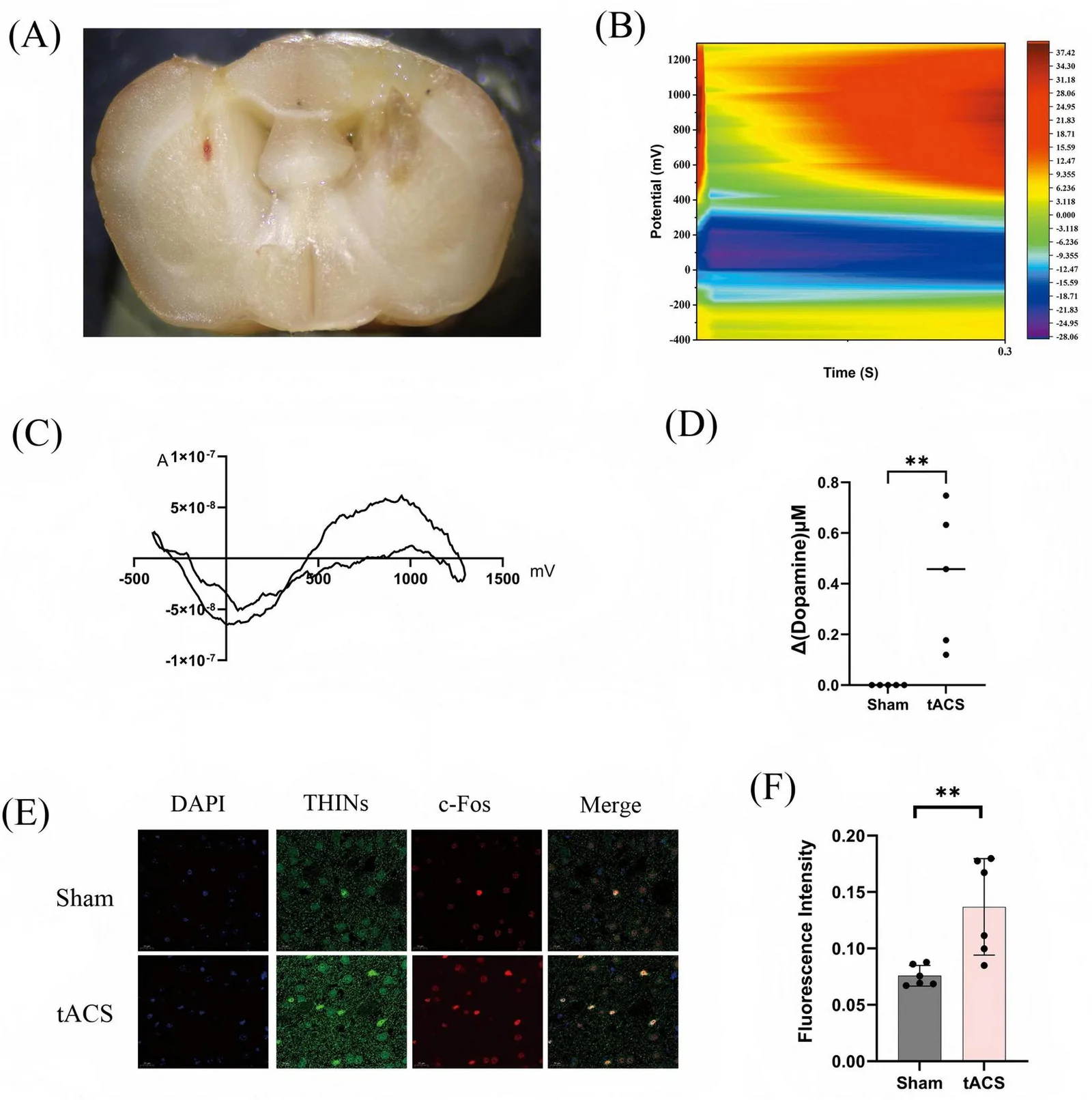
Transcranial alternating current stimulation (tACS)-induced dopamine efflux and neuronal activation in the striatum. **(A)** Schematic illustration of the implantation sites of FSCV carbon-fiber microelectrodes in striatum (red diamond); **(B)** Corresponding three-dimensional pseudo-color plot (current–voltage–time) from the recording shown, highlighting the characteristic dopamine oxidation peak at approximately +0.6 V; **(C)** Representative time course of dopamine efflux recorded in the striatum following a single bout of tACS, obtained at a holding potential of +0.62 V versus Ag/AgCl; **(D)** Quantitative analysis of peak dopamine concentration changes recorded in the striatum after tACS over M1. Data are presented as mean ± SEM (*n* = 5 mice). *p < 0.05, **p < 0.01; **(E)** Representative immunofluorescence images of the striatum in Sham and tACS-treated mice, showing DAPI-labeled nuclei (blue), THINs (TH, green), and c-Fos (red), as well as their co-localization; **(F)** Quantification of activated THINs (c-Fos/TH) in the striatum. Neuronal activity was assessed as the ratio of the integrated optical density of c-Fos immunofluorescence to the corresponding area. Data are presented as mean ± SEM (*n* = 6 per group). Data are presented as mean ± SEM, **p < 0.01 versus Sham.

Using FSCV, we observed a rapid, transient rise in extracellular dopamine concentration in the dorsolateral striatum immediately following a single tACS session. Peak dopamine release was significantly higher in the tACS group than in the sham controls (*p* = 0.0079; *U* = 0, Figure 2D). Immunofluorescence staining revealed a marked increase in the number of activated c-Fos and THINs in the striatum of tACS-treated mice compared with sham controls [(*p* = 0.0065, *t* = 3.420, df = 10, 95% CI [0.0213, 0.1009]); Figure 2F].

### 3.3 Optogenetic activation of THINs alone is sufficient to improve motor performance and enhance beta oscillations

**FIGURE 3:**
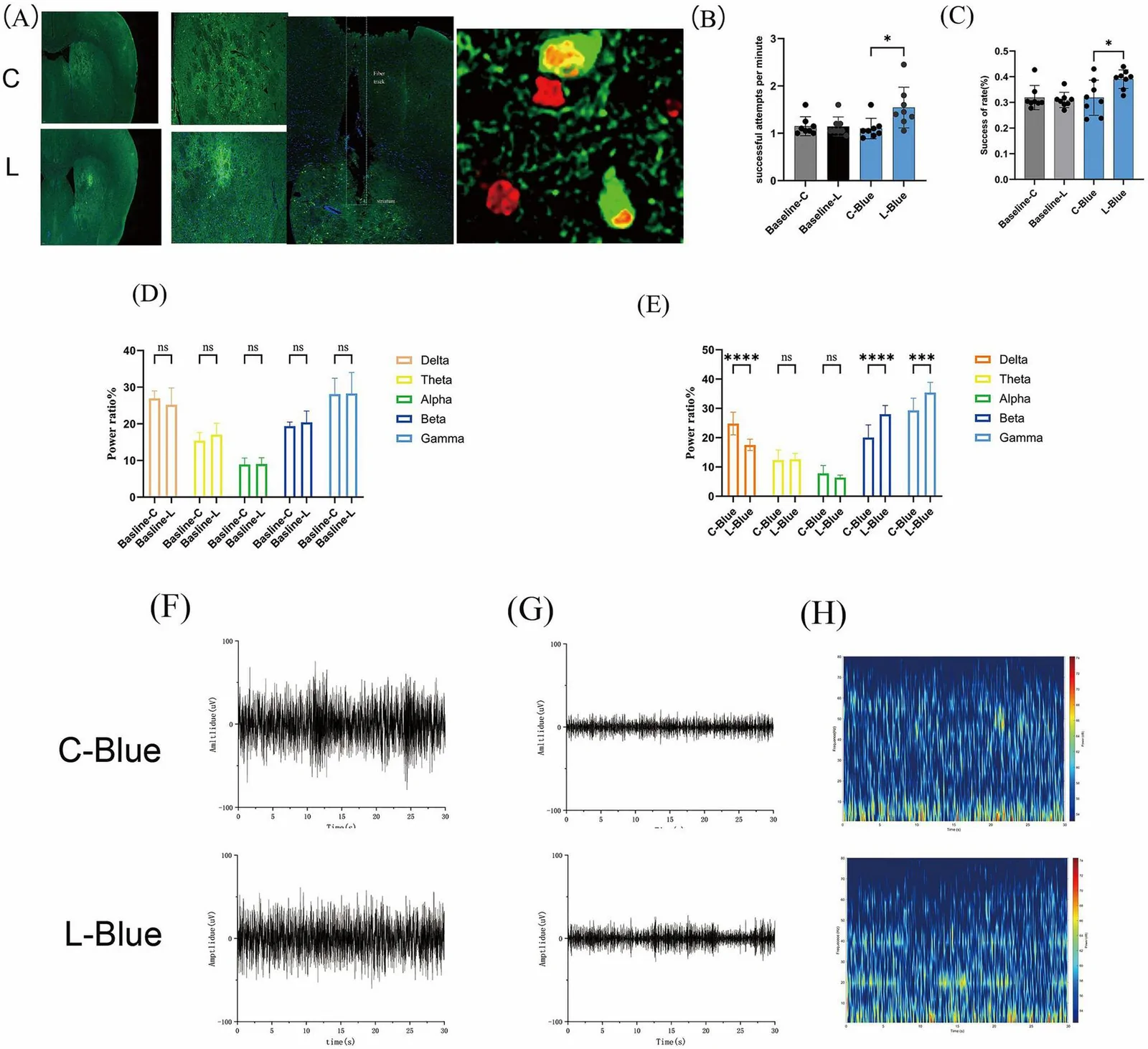
Behavioral and EEG effects of optogenetic stimulation of striatal THINs. **(A)** Coronal sections showing EGFP expression following control rAAV-EGFP (C) and rAAV-ChR2-EYFP (L) injection, demonstrating specific fusion-protein expression in striatal THINs Left: whole overview; right: higher magnification of boxed regions in striatal; **(B)** The successful attempts per minute pre- and post-optogenetic stimulation; Baseline success rates were recorded prior to any intervention (C-Sham, L-Sham); Post-intervention success rates were assessed on day 7 following either control C-Blue or L-Blue (*n* = 8 per group); **(C)** Success rates pre- and post-optogenetic stimulation; Baseline success rates were recorded prior to any intervention (C-Sham, L-Sham); Post-intervention success rates were assessed on day 7 following either control C-Blue or L-Blue (*n* = 8 per group). **(D)** Bar graphs showing the quantitative comparison of baseline relative power in the δ, θ, α, β, γ between the two groups. **(E)** Bar graphs showing the quantitative comparison of relative power in the δ, θ, α, β, γ between the two groups during light stimulation. **(F)** Representative raw traces; **(G)** β-band EEG; **(H)** Time–frequency representations, under blue light optogenetic stimulation. Upper panels: control mice injected with rAAV-EGFP; lower panels: rAAV-ChR2-EYFP. Data are presented as mean ± SEM. *p < 0.05, ***p < 0.001, ****p < 0.0001 compared with the Sham group.

To validate TH promoter specificity, we performed quantitative colocalization in striatal sections from a validation cohort (*n* = 3), Of 119 Chr2-EGFP-positive neurons, 74.79% (89/119) co-expressed TH (Figure 3A), confirming specific transduction of striatal THINs. Compared with the C-Blue group, the L-Blue group injected with the Chr2 virus showed a significantly higher successful attempts per minute (*p* = 0.0107, Figure 3B) and success rate in the single-pellet reaching task following 470-nm blue-light stimulation (*p* = 0.0207, Figure 3C). At baseline, there was no significant difference in the δ(26.95% ± 2.02% vs. 25.22% ± 4.57%, *p* = 0.2948, *t* = 1.005, 95% CI [−1.541, 5.005]), θ(15.41% ± 2.24% vs. 17.04% ± 3.11%, *p* = 0.3256, *t* = 0.990, 95% CI [4.897, 1.648]), α(8.94% ± 1.74% vs. 9.06% ± 1.69%, *p* = 0.9411, *t* = 0.0741, 95% CI [−3.395, 3.151]), β(19.38% ± 1.10% vs. 20.41% ± 3.07%, *p* = 0.5322, *t* = 0.6278, 95% CI [−4.303, 2.243]), γ(28.13% ± 4.32% vs. 28.27% ± 5.73%, *p* = 0.9286, *t* = 0.0899, 95% CI [−3.420, 3.125]) band intensity between the group subsequently assigned to baseline-C and the group baseline-L (*p* > 0.05; Figure 3D). Following activation of THINs, EEG activity showed a marked increase β(28.03% ± 2.95% vs. 20.05% ± 4.31%, *p* < 0.0001, *t* = 5.063, 95% CI [−11.132, −4.836]), γ(35.40% ± 3.51% vs. 29.32% ± 4.16%, *p* = 0.0003, *t* = 3.856, 95% CI [−9.220, −2.933]), and a marked reduction in relative power δ(17.54% ± 1.95% vs. 24.81% ± 3.86%, *p* < 0.0001, *t* = 4.614, 95% CI [4.128, 10.415]), compared to the C-Blue groups (Figure 3E). The corresponding EEG waveforms and time–frequency plots were also altered, and the EEG and time–frequency plots revealed a clear increase in β power (Figures 3F–H).

### 3.4 Optogenetic inhibition of THINs via eNpHR3.0 was sufficient to reduce the successful attempts per minute and suppress beta oscillations

**FIGURE 4:**
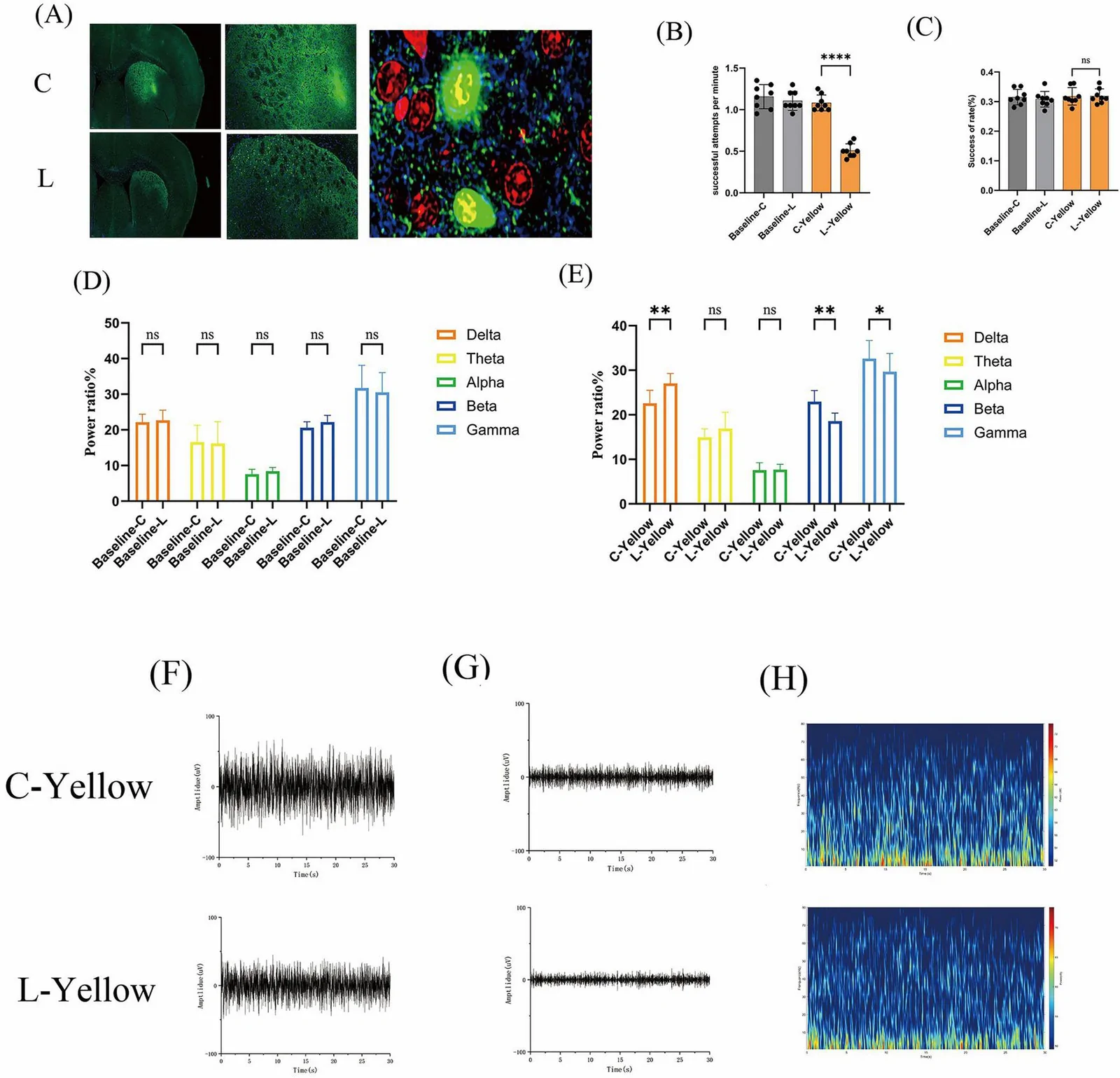
Behavioral and EEG effects of optogenetic inhibition of striatal THINs. **(A)** Coronal sections showing EGFP expression following control rAAV-EGFP (C) and rAAV-eNpHR3.0-EYFP (L) injection, demonstrating specific fusion-protein expression in striatal THINs Left: whole overview; right: higher magnification of boxed regions in striatal; **(B)** The successful attempts per minute pre- and post-optogenetic stimulation; Baseline success rates were recorded prior to any intervention (C, L); Post-intervention success rates were assessed on day 7 following either control C-Yellow or L-Yellow (*n* = 8 per group). **(C)** Success rates pre- and post-optogenetic stimulation; Baseline success rates were recorded prior to any intervention (C, L); Post-intervention success rates were assessed on day 7 following either control C-Yellow or L-Yellow (*n* = 8 per group); **(D)** Bar graphs showing the quantitative comparison of baseline relative power in the δ, θ, α, β, γ between the two groups. **(E)** Bar graphs showing the quantitative comparison of relative power in the δ, θ, α, β, γ between the two groups during light stimulation; **(F)** Representative raw traces; **(G)** β-band EEG **(H)** Time–frequency representations, under yellow light optogenetic stimulation. Upper panels: control mice injected with rAAV-EGFP; lower panels: rAAV-eNpHR3.0-EYFP. Data are presented as mean ± SEM. *p < 0.05, **p < 0.01, ****p < 0.0001 compared with the Sham group.

To validate TH promoter specificity, we performed quantitative colocalization in striatal sections from a validation cohort (*n* = 3). Of 121 eNpHR3.0-EGFP-positive neurons, 71.07% (86/121) co-expressed TH (Figure 4A), confirming specific transduction of striatal THINs. Compared with the C-Yellow group, the L-Yellow group injected with the eNpHR3.0 virus showed a significantly lower number of successful attempts per minute in the single-pellet reaching task following 560-nm yellow-light stimulation (*p* < 0.0001, Figure 4B), whereas no statistically significant reduction in success rate was detected (*p* = 0.9548, Figure 4C). At baseline, there was no significant difference in the δ(22.14% ± 2.26% vs. 22.69% ± 2.84%, *p* = 0.7825, *t* = 0.2771, 95% CI [−4.446, 3.361]), θ(16.55% ± 4.71% vs. 16.19% ± 6.11%, *p* = 0.8547, *t* = 0.1838, 95% CI [−3.544, 4.263]), α(22.14% ± 2.26% vs. 22.69% ± 2.84%, *p* = 0.6680, *t* = 0.4307, 95% CI [−4.746, 3.060]), β(20.60% ± 1.68% vs. 22.20% ± 1.87%, *p* = 0.4164, *t* = 0.8176, 95% CI [−5.504, 2.303]), γ(31.74% ± 6.38% vs. 30.52% ± 5.53%, *p* = 0.5352, *t* = 0.6232, 95% CI [−2.684, 5.123]) band intensity between the group subsequently assigned to baseline-C and the group baseline-L (*p* > 0.05; Figure 4D). Following inhibition of THINs, EEG activity showed a marked reduction β(18.59% ± 1.81% vs. 22.94% ± 2.51%, *p* = 0.0026, *t* = 5.063, 95% CI [1.572, 7.127]), γ(29.70% ± 4.08% vs. 32.60% ± 4.09%, *p* = 0.0406, *t* = 2.086, 95% CI [0.1275, 5.6820]), and increase in relative power δ(27.06% ± 2.21% vs. 22.59% ± 2.93%, *p* = 0.0020, *t* = 3.215, 95% CI [−7.254, −1.699]), compared to the C-Yellow groups (Figure 4E). The corresponding EEG waveforms and time–frequency plots were also altered, and the EEG and time–frequency plots revealed a clear reduction in β power (Figures 4F–H).

## 4 Discussion

In this study, we examined whether 20 Hz tACS over M1 is associated with changes in post-anesthesia motor performance and related striatal correlates. We demonstrated that repeated 20 Hz tACS significantly enhances skilled reaching performance (Figures 1F, G), an effect accompanied by a selective increase in β-band (12–30 Hz) oscillatory power (Figures 1H–L) and elevated striatal dopamine release (Figures 2A–D), We found that 7-days 20 Hz tACS over M1 robustly engaged striatal THINs, as shown by a significant increase in the number of c-Fos^+^ THINs following stimulation (Figures 2E, F). Notably, the degree of THIN activation correlated with both β-power and motor performance, suggesting a correlational bridge that warrants further causal testin.

The selective coupling between β-band oscillations and motor improvement observed here provides novel insight into the role of cortical–striatal synchrony in action regulation. Classically, β-activity has been associated with maintaining the motor status quo and with antidromic motor control; however, accumulating evidence suggests that β-power also encodes motor vigor and incentive drive (Savoie et al., 2019; Hoy et al., 2024; Pierrieau et al., 2026). Our findings demonstrate that direct THIN activation elevate β-band power while simultaneously increasing the rate of successful reaches and altering reaching accuracy (Figures 3B, C). This selective enhancement of movement frequency, rather than fine motor precision, implies that β-synchrony may gate the velocity or motivational gain of motor output (Figures 3D–H). In the opposing manipulation, THIN inhibition reduced β-power (Figures 4D–H) and selectively slowed the pace of successful reaches (Figures 4B, C), aligning with reports that diminished β-oscillations are associated with bradykinesia (Jiang et al., 2024; Kehnemouyi et al., 2021; Schwerdt et al., 2020). These data collectively suggest that β-band activity serves as a physiological mediator translating motivational signals into motor vigor. Whether its enhancement directly counteracts motor slowing requires further interventional studies, particularly in dopamine-deficient states.

At the neurochemical level, THINs are ideally positioned to integrate this mechanism, as key relay stations regulated primarily by dopamine, which enhances their plateau potentials and excitability (Ibáñez-Sandoval et al., 2015; Siegel et al., 2009), THINs are positioned to link dopaminergic signaling with β-band synchrony modulation, although the direct conversion step remains to be definitively established. This interpretation is consistent with a working model in which cortical stimulation, dopamine release, THIN activation, and β-synchrony enhancement are convergingly associated with increased motor drive. However, we acknowledge that our current data do not fully establish this as a complete serial causal chain, because we did not directly test whether THIN activity is necessary during tACS delivery (e.g., by inhibiting THINs concurrently with tACS) or whether dopamine receptor blockade abolishes the tACS effect. Thus, while the converging evidence is strong, the definitive causal sequence requires further experimental validation (Kaminer et al., 2019).

Our repeated daily stimulation paradigm appears critical for the durability of these effects. While single-session β-band stimulation has shown limited efficacy on motor learning or memory in older adults (Rumpf et al., 2019; Puri et al., 2021), the 7-days protocol employed here may cumulatively promote sustained synaptic plasticity within striatal circuits, leading to persistent THIN engagement and stable β-power enhancement. These results align with growing evidence that frequency-specific neuromodulation can improve neurological function in neurodegenerative conditions, and underscore the importance of repeated intervention protocols for achieving clinically meaningful outcomes (Cantoni et al., 2025; Ye et al., 2025; Sánchez-Garrido Campos et al., 2025). A critical methodological consideration is that tACS and FSCV recordings were performed under sevoflurane anesthesia (2.3%), while behavioral and EEG assessments were conducted 3 min after awakening. Sevoflurane is known to suppress endogenous cortical and striatal dynamics (Zhang et al., 2023), depress dopamine release (Bao et al., 2025), and alter β-band oscillations (Jiang et al., 2019). Therefore, the observed effects may partially reflect enhanced post-anesthetic recovery rather than, or in addition to, a reversal of motor changes specifically attributable to aging. Our current design, lacking a young control group, cannot distinguish these possibilities. Moreover, individual variability in emergence times from anesthesia likely contributed to variance in early post-awakening motor performance. Although we standardized the 3-min post-awakening testing window, residual anesthetic effects on motor vigor and EEG signatures cannot be fully excluded. Furthermore, the absence of a young control group means that our data cannot directly distinguish age-specific effects from general anesthesia-related recovery effects. Future studies employing awake tACS delivery and including young control groups are necessary to dissociate these confounds and to determine the extent to which the observed effects are specific to the aged brain versus reflecting enhanced recovery from anesthesia. In addition, the relatively small sample size (*n* = 5–8 per group) may limit the statistical power to detect subtle effects. The exclusive use of male mice, while experimentally justified to avoid estrous cycle variability, limits the generalizability of our findings to females. Given known sex differences in striatal dopamine signaling and motor function, future studies including female mice are needed to determine whether the correlational pattern among dopamine, THIN activity, and β-band power observed here extends to both sexes.

Taken together, our findings indicate that 20 Hz tACS acts on the aged striatum through dopaminergic enhancement of THIN-dependent facilitation of β-band synchrony, ultimately boosting motor drive. This multilevel mechanism not only clarifies how rhythmic cortical stimulation translates into behavioral improvement but also establishes THINs and β-oscillations as promising therapeutic targets for alleviating motor deficits in aging and related neurodegenerative disorders.

## 5 Conclusion

In summary, this study provides converging evidence that 20 Hz tACS over M1 is associated with improved post-anesthesia motor performance, associated with striatal dopamine release, THIN engagement, and β-band synchrony enhancement. Our findings reveal that THINs function as gain modulators that convert dopaminergic signals into enhanced β-oscillatory power, thereby boosting motor drive thereby boosting motor drive, while no statistically significant reduction in accuracy was detected in the inhibition experiment. These results suggest that frequency-specific neuromodulation of the cortical-striatal circuit may represent a promising strategy for enhancing motor function in aged populations. While the long-term durability and safety of this intervention remain to be fully characterized, the multilevel mechanistic framework presented here provides a foundation for developing targeted, non-invasive therapeutic approaches for improving post-anesthesia motor performance in aged mice. Whether this effect generalizes to age-related motor deficits in the awake state, or to neurodegenerative conditions, warrants further investigation.

## Data Availability

The original contributions presented in this study are included in this article/supplementary material, further inquiries can be directed to the corresponding author.
